# How digital lifestyles transform women’s exercise participation: a mixed methods study from China

**DOI:** 10.3389/fpsyg.2026.1896888

**Published:** 2026-09-03

**Authors:** Jing Huang, Kailin Wu, Qinghao Guan, Qing Liu, Yifan Zuo

**Affiliations:** 1 School of Physical Education, Hunan University of Technology, Zhuzhou, China; 2 Department of Basic Courses, Hunan Polytechnic of Water Resources and Electric Power, Changsha, China; 3 Department of Communication and Media Research, University of Zurich, Zurich, Switzerland; 4 School of Physical Education and Sports Science, South China Normal University, Guangzhou, China; 5 School of Sports, Shenzhen University, Shenzhen, China

**Keywords:** digital lifestyles, fitness habits, gender and technology, health equity, women’s exercise participation

## Abstract

**Introduction:**

Digital technologies are transforming women’s access to physical exercise, yet empirical evidence from non-Western contexts remains limited. This study examines whether digital lifestyle is associated with women’s exercise participation in China and whether this association differs across socio-demographic groups.

**Methods:**

We used a mixed-methods design. Study 1 analysed data from 4,469 women in the 2021 Chinese General Social Survey (CGSS) using regression and heterogeneity analyses. A composite Digital Lifestyle Index was constructed from internet-use indicators. Study 2 involved 12 in-depth interviews analysed with grounded theory to uncover underlying mechanisms.

**Results:**

Digital lifestyle was positively associated with exercise frequency (*β* = 0.568, *p* < 0.001), robust to controls. This association was significantly stronger for women with non-agricultural hukou (*β* = 0.654, *p* < 0.001) and urban residents (*β* = 0.470, *p* < 0.01), but did not vary by age, marital status, education, or region. Qualitative findings show that digital platforms create flexible access, foster social support, and build health knowledge, gradually internalising exercise into daily habits.

**Discussion:**

Digital lifestyles can empower women’s exercise participation but benefits are unevenly distributed across urban–rural and hukou divides. Policies should address infrastructure and literacy gaps to ensure equitable access, extending feminist and field theories to the digital health context.

## 1 Introduction

As women’s liberation and the pursuit of rights have progressed, increasing attention has been given to women’s participation in physical exercise. Engaging in sports and fitness activities not only enhances women’s physical and mental well-being and overall life satisfaction but also fosters self-worth and personal development. However, structural barriers have long hindered women’s participation in physical exercise (Henderson et al., 1989), particularly due to the gendered division of labor, which limits their time, space, energy, and awareness to prioritize their health needs and leisure activities. Fortunately, digital technologies, with their capacity to transcend spatial and temporal constraints, enable personalized fitness guidance and real-time health monitoring to overcome these limitations, allowing data to be permanently stored and widely disseminated in digital form. For women, digitalization is breaking traditional gender role constraints, overcoming time and space limitations, and providing greater opportunities for physical exercise. The growing popularity of fitness applications such as Sweat and EvolveYou has lowered the barriers to participation in physical exercise. It is estimated that the penetration rate of digital fitness in the United States is projected to reach 57.63% by 2025 and is expected to rise to 65.04% by 2029 (Statista, 2024).

Previous studies have demonstrated that digital lifestyles play three key roles in being associated with women’s participation in physical activities. First, digital lifestyles serve as a catalyst for are associated with increased women’s engagement in sports and fitness activities. With the rapid advancement of internet technologies and the rise of the digital era, women’s participation rates in physical exercise have significantly increased (Kross et al., 2021; Frison and Eggermont, 2020). Second, digital lifestyles create diverse opportunities for women, providing channels for entertainment, social engagement, and sports-related consumption. This multifaceted role enriches women’s daily lives and facilitates their comprehensive participation in physical activities (Huberty et al., 2013). Moreover, women can engage in entertainment and online consumption, influencing their preferences and choices, which collectively shape their participation in physical activities (Andersen and Bakken, 2019; Ferry, 2014; Lin et al., 2021). Seeing women on digital platforms showcase their athletic abilities and prioritize overall wellness can serve as a powerful inspiration for other women to engage in sports and improve their own health. This positive representation shifts the focus away from the pressure to meet unrealistic beauty standards, allowing women to pursue fitness goals that truly benefit their physical and mental well-being (Trolan, 2013). While researchers have examined the positive impact of digital lifestyles on exercise participation, there is still a lack of research specifically focusing on, at the micro level, on how digital lifestyles are related to women’s fitness behaviors. On the other hand, large-scale survey studies are scarce, and the internal mechanisms by which digital lifestyles reshape women’s exercise habits remain underexplored.

The current research employs a mixed method. In Study 1, we utilize data from the Chinese General Social Survey 2021(CGSS) to analyze the association between digital lifestyles and women’s exercise habits. The second study applies qualitative methods to explore the underlying mechanisms through which digital lifestyles influence women’s physical activity. This research holds significant practical implications for deepening the understanding of how digital lifestyles relate to exercise behaviors, promote women’s health, and advance health equity for women.

## 2 Literature review

### 2.1 Digitalization and physical exercises

The rapid development of digital technology has profoundly influenced the accessibility and overall experience of physical exercise. By reviewing previous research, this paper explores the impact of digitalization on exercise, focusing on several key aspects: (a) the relationship between digitalization and exercise behavior; (b) changes in exercise motivation; (c) innovations in exercise approaches; and (d) the potential negative effects of digitalization on exercise.

#### 2.1.1 Digitalization and exercise behavior

The widespread adoption of digital technology has provided users with convenient access to sports and fitness-related information. Digitalization enables individuals to easily find fitness guidance, exercise techniques, and health advice, thereby promoting self-managed physical activity (Sevilmis et al., 2023). Additionally, Loprinzi et al. (2021) found that digital tools, such as fitness applications (e.g., 8fit), video platforms (e.g., YouTube), and social media (e.g., Instagram), offer users diverse exercise resources, making it easier to engage in regular physical activity. As a public network platform, digital tools also allow users to establish and participate in online fitness communities. These virtual communities enhance users’ exercise motivation through online interactions. As De Santis et al. (2021) noted, exercise sharing on social media, online fitness challenges, and team-based interactions can significantly increase users’ workout frequency and duration. Such platforms not only provide features for tracking and sharing fitness data (Gui et al., 2017), but also create a social environment where users can monitor, motivate, and encourage one another.

#### 2.1.2 Digitalization and changes in exercise motivation

Digitalization has effectively transformed individuals’ motivation to engage in physical activity through external incentives, goal setting, and the enhancement of self-efficacy. Digital technology—particularly smart devices and fitness applications—motivates users by quantifying exercise performance. These tools measure metrics such as steps taken, distance traveled, duration of workouts, heart rate, caloric expenditure, and even sleep quality. By presenting this data in real-time or through detailed summaries, they provide concrete evidence of progress and performance. This quantification allows users to set specific goals, track improvements, and stay motivated to maintain or increase their activity levels (Gilmore, 2016). A study by Nuss et al. (2021) found that exercise data visualization tools, such as step tracking and calorie expenditure metrics, allow users to better understand their exercise progress, thereby strengthening their extrinsic motivation. Additionally, the goal-setting and progress-tracking features provided by digital tools offer users a clearer direction and structured exercise plans. In terms of enhancing self-efficacy, digitalization provides a vast array of online courses and tutorials, enabling users to independently learn and develop various exercise skills (Domin et al., 2021).

#### 2.1.3 Digitalization and innovations in exercise approaches

Digitalization has led to the emergence of numerous online fitness platforms, which offer diverse workout programs through live streaming and on-demand videos. Online yoga classes, home workout programs, and similar digital offerings provide fitness enthusiasts with greater flexibility, a trend that became particularly widespread during the COVID-19 pandemic (Carraça et al., 2021; Zuo et al., 2021). These online platforms not only eliminate the constraints of traditional gym facilities but also increase the diversity of exercise options. Moreover, digitalization has facilitated the gamification of exercise methods. For instance, fitness games based on Augmented Reality and Virtual Reality enable users to achieve their workout goals while engaging in entertaining activities (Mouatt et al., 2020). Tuveri et al. (2016) observed that interactive fitness games, such as *Beat Saber*, which integrate physical movement with gaming elements, have successfully attracted individuals who are less inclined toward traditional workout routines, effectively increasing their participation in physical activity through gamification.

Despite the positive role of digitalization in promoting physical exercise, it also brings certain negative effects. Kaur et al. (2020) pointed out that users may develop excessive dependence on fitness applications and smart devices, leading to a neglect of natural bodily feedback. Moreover, an overemphasis on exercise data and virtual fitness communities may weaken intrinsic motivation for physical activity, ultimately affecting long-term exercise adherence. Additionally, attention should be given to the issue of information overload. Information overload occurs when the amount of data presented exceeds an individual’s capacity to process and interpret it effectively, leading to confusion and diminished engagement (Belabbes et al., 2023). This was also elaborated by Rice and Sara (2019), who suggests that users may experience decision paralysis, struggling to determine the most suitable workout methods and programs when faced with an overwhelming variety of fitness content. This could ultimately reduce their engagement in physical exercise.

While these drawbacks underscore the complexities associated with integrating technology into fitness routines, they also open an important avenue for further inquiry. Specifically, understanding how digital tools influence exercise engagement requires looking beyond generalized concerns of information overload and dependence on devices. Factors like social norms, personal goals, and individual contexts may shape users’ experiences differently, particularly for groups such as women. By examining these nuances, researchers can gain insights into how digitalization both empowers and potentially constrains exercise behavior across diverse populations.

For women, what is the intrinsic relationship between digital lifestyles and exercise behavior? Does this relationship vary across different factors such as household type, urban–rural classification, and marital status? These questions have yet to be empirically examined in existing research.

Therefore, this study proposes the following hypotheses:

*Hypothesis 1:* There may be a positive correlation between digital lifestyle and women's physical exercise behavior.

*Hypothesis 2*: the positive association between digital lifestyle and women's exercise behavior may differ significantly by household type, urban-rural residence, and educational attainment.

### 2.2 Theoretical framework

#### 2.2.1 Field Theory

Field Theory, proposed by Pierre Bourdieu, provides a foundational framework for analyzing social practices and power relations through three core concepts: field, capital, and habitus. A field denotes a network of objective relations between positions, each field operating with its own logic and competitive rules. Capital manifests primarily in four forms—economic, cultural, social, and symbolic—serving as the resources enabling actors to compete within a field, with symbolic capital being the most potent and convertible form. Habitus is an open system of dispositions that helps actors understand and strategically navigate a field’s rules. Applying this framework to digital lifestyles and women’s exercise habits illuminates how women reshape their fitness behaviors within a multi-layered social, cultural, and economic context.

#### 2.2.2 Feminist Sports Theory

Feminist Sports Theory offers a critical lens for analyzing and deconstructing gender inequalities, power dynamics, and cultural norms within sports (Hargreaves, 1986). Integrating feminist theory with sports research, this perspective examines how sports reflect, reinforce, or challenge societal gender structures and explores pathways to gender equality and women’s empowerment through sport (Hood-Williams, 1995). Applying Feminist Sports Theory to examine the impact of digital lifestyles on women’s exercise habits allows for a deeper understanding of how digital lifestyles shape women’s participation in sports. Additionally, it helps uncover the underlying gender power dynamics and the potential for women’s empowerment within digitalized fitness practices.

## 3 Research design overview

This study employs a mixed method to examine the impact of digital lifestyles on women’s exercise habits and the underlying mechanisms driving these changes. The research design consists of two main components (see Table 1). Study 1 is based on data from the Chinese General Social Survey (CGSS) and utilizes a multiple linear regression model to explore the influence of digital lifestyles on women’s exercise behavior. It also examines differences among women from various social groups. Study 1 aims to validate the hypothesis that digital lifestyles affect women’s participation in physical exercise and to identify potential variations in this effect among women with different social characteristics.

**Table 1:** Research design.

Study	Materials	Data size	Method	Targets
Study 1	Chinese General Social Survey (CGSS)	4,469	Multiple linear regression model	“Digital lifestyle may promote women’s physical activity, yet such promotive effects are unlikely to be uniform and may be moderated by key social factors such as household type, geographic location, and education.”
Study 2	In-depth interview	12	Grounded Theory	The mechanism of how digital lifestyles influence women’s exercise participation

Building on these findings, the authors further investigate the underlying mechanisms driving this influence, leading to Study 2. Given the lack of theoretical studies on these mechanisms, Study 2 employs in-depth interviews with women to collect and analyze qualitative data. Using grounded theory, this study explores how digital lifestyles shape women’s exercise behaviors and identifies the key factors involved in this process.

Through this research design, the study seeks to provide a comprehensive understanding of how digital lifestyles transform women’s exercise habits. The findings aim to offer theoretical insights for promoting women’s health and advancing gender health equity.

## 4 Study 1

### 4.1 Methods

#### 4.1.1 Data source

CGSS aims to collect longitudinal data at the individual, household, and community levels to track changes in China’s society, economy, population, education, and health. This dataset serves as a foundational resource for academic research and public policy analysis. CGSS covers 25 provinces, municipalities, and autonomous regions, with a target sample size of 16,000 households, thus, it is one of the most representative databases on individual digital engagement at an international level (Wang and Wang, 2024). The survey includes all family members within sampled households and was officially launched in 2010, with follow-up surveys conducted every 2–3 years. The data used in this study are drawn from the 2021 wave of the CGSS, which contains demographic information, sports participation data, and digital usage-related variables. This study focuses on female participants, and after removing missing and abnormal values, the final valid sample consists of 4,469 individuals. The CGSS data used in this study were obtained from a publicly available dataset approved by the Peking University Biomedical Ethics Review Committee (IRB00001052-14010), which applies to all survey waves and does not vary with time. All participants in the CGSS gave informed agreement to participate in the survey in accordance with ethical guidelines at the time of data collection.

#### 4.1.2 Variable description

##### 4.1.2.1 Dependent variable

Exercise frequency was measured by the question: “In the past year, how frequently did you engage in physical exercise during your leisure time?” Responses were recorded on a 5-point Likert scale (1 = never, 5 = very frequently), with higher scores denoting greater exercise frequency.

##### 4.1.2.2 Independent variable

The Digital Lifestyle Index was constructed as a composite measure using four items from the CGSS 2021 questionnaire: (a) frequency of internet use in the past year; (b) frequency of internet use during leisure time; (c) whether the internet is the primary source of information; and (d) whether the respondent has used the internet in the past 6 months. To validate the appropriateness of aggregating these four indicators into a composite index, we conducted structural validity tests. The Bartlett’s test of sphericity yielded a chi-square statistic of 17,005.278 (df = 6, *p* < 0.001), indicating substantial intercorrelations among the items and supporting the suitability of factor analysis. The Kaiser–Meyer–Olkin (KMO) measure of sampling adequacy was 0.850, indicating excellent sampling adequacy (>0.8). Further principal component analysis (PCA) revealed that the first principal component had an eigenvalue of 3.423, explaining 85.59% of the total variance. The factor loadings for the four items were 0.5094, 0.5129, 0.4755, and 0.5013, respectively, all demonstrating good performance and suggesting that a single factor adequately captured the common variance among the four indicators. Based on these results, we used the first principal component score (PC₁) as the foundation for the Digital Lifestyle Index. Specifically, the first principal component score was computed as:

$${\mathrm{PC}}_{1}=0.5094\times Z_{1}+0.5129\times Z_{2}+0.4755\times Z_{3}+0.5013\times Z_{4}$$

where *Z*₁ through *Z*₄ represent the *Z*-score standardized values of the four original indicators. After calculating PC₁ scores for each respondent, we further applied min-max normalization to compress the scores to a 0–1 range, generating the final “Digital Lifestyle Index.” Higher values indicate a higher degree of digital lifestyle engagement.

##### 4.1.2.3 Control variables

We included a range of control variables, which were grouped into three categories: individual characteristics (age, marital status, educational level), social characteristics (hukou status, self-perceived social class, economic status, personal income, household economic status), and regional characteristics (urban–rural classification, economic region).

The measurement of relevant variables and their descriptive statistics are presented in Table 2.

**Table 2:** Measurement and descriptive statistics of relevant variables.

Variable	Sample size	Mean	Standard deviation	Min	Max	Description
Digital lifestyle	4,277	0.651	0.411	0	1	Composite index
Frequency of internet use in the past year	4,467	3.320	1.662	1	5	Responses were recorded on a 5-point scale (1 = lowest frequency, 5 = highest frequency)
Frequency of internet use during leisure time	4,453	3.542	1.808	1	5	Responses were recorded on a 5-point scale (1 = lowest frequency, 5 = highest frequency)
Whether the internet is the primary source of information	4,297	0.622	0.485	0	1	No partner = 0, with partner = 1
Whether the respondent has used the internet in the past 6 months	4,465	0.699	0.459	0	1	No partner = 0, with partner = 1
Exercise frequency	4,459	2.757	1.621	1	5	Scale from 0 to 4, indicating increasing exercise frequency
Age	4,469	51.16	17.26	18	99	Numeric
Age group	4,469	0.458	0.498	0	1	Age was grouped as follows: women (<55 = 0, ≥55 = 1); men (<60 = 0, ≥60 = 1).”
Household type	4,417	0.382	0.486	0	1	Agricultural household = 0, non-agricultural household = 1
Marital status	4,469	0.743	0.437	0	1	No partner = 0, with partner = 1
Education level	4,460	0.194	0.396	0	1	Basic education = 0, higher education = 1
Social class	4,294	4.350	1.852	1	10	Scale from 0 to 10, indicating increasing social class
Economic status	4,358	3.700	0.896	1	5	Scale from 0 to 5, indicating increasing economic status
Personal income	3,968	7.237	4.547	0	12.47	Logarithm of annual personal income
Household economic status	4,357	2.568	0.757	1	5	Scale from 0 to 5, indicating increasing household economic status
Urban/rural	4,469	0.572	0.495	0	1	Rural = 0, urban = 1
Economic region	4,469	1.132	0.800	0	2	Western = 0, central = 1, eastern = 2

#### 4.1.3 Model settings

To examine the effect of digital lifestyle on women’s exercise frequency (H1), we constructed the following multiple linear regression model as our baseline specification (see Equation 1):

$$\mathrm{PA}F_{i}=\beta_{0}+\beta_{1}\cdot {\mathrm{DL}}_{i}+\Sigma \gamma_{j}\cdot C_{\mathrm{ji}}+\varepsilon_{i}$$ (1)

where the subscript *_i_* indexes individual respondents; PAF*_i_* denotes exercise frequency, measured on a 5-point ordinal scale (1 = lowest, 5 = highest); DL*_i_* is the Digital Lifestyle Index, a continuous variable ranging from 0 to 1; *C_ji_* represents a vector of control variables, including age, hukou (household registration), marital status, educational level, social class, economic status, logarithm of personal income, household economic status, urban–rural classification, and economic region; and *ε_i_* is the random error term.

To test whether the relationship between digital lifestyle and exercise frequency varies significantly across different socio-demographic groups (H2), we employed Chow tests by incorporating interaction terms between the grouping variables and the digital lifestyle index. Taking household registration as an illustrative example, the model was specified as follows (see Equation 2)

$$\mathrm{DL}=\beta_{0}+\beta_{1}{\mathrm{PAF}}_{i}+\beta_{2}{\mathrm{PAF}}_{i}M+\beta_{3}M+\beta_{n}C_{n}+\varepsilon$$ (2)

where PAF denotes exercise frequency; DL is the Digital Lifestyle Index; Hukou is a binary indicator (1 = non-agricultural, 0 = agricultural); and *C_ji_* represents the vector of control variables. The interaction term coefficient, *β*_3_, captures whether the association between digital lifestyle and exercise frequency differs significantly between women with agricultural and non-agricultural household registration. A statistically significant *β*_3_ would indicate significant between-group heterogeneity. The same model specification was applied to the other grouping variables, including age group, marital status, educational level, urban–rural classification, and economic region, with Hukou replaced by the corresponding grouping variable.

A methodological caveat should be noted. The data used in this analysis are drawn from the 2021 CFPS, a cross-sectional survey. Therefore, the regression models presented above reflect statistical associations between digital lifestyle and women’s exercise frequency rather than causal effects. All coefficient estimates are interpreted as indicating positive or negative correlations after controlling for covariates, and no causal direction should be inferred from these findings.

### 4.2 Findings

#### 4.2.1 Digital lifestyle and exercise frequency among women

To preliminarily examine the distributional differences in digital lifestyle and exercise frequency across women with different socio-demographic characteristics, we first conducted univariate descriptive statistics and difference tests for each subgroup. The results are presented in Table 3.

**Table 3:** Digital lifestyle and exercise frequency among women of different demographic groups.

Variables	Groups	*N*	Digital lifestyle		*N*	Physical exercise frequency	
			Mean (SD)	*t*/*F*		Mean (SD)	*t*/*F*
Household type	Non-agricultural household = 1	1,659	0.734 (0.371)	−10.67***	1,684	3.269 (1.540)	−17.36***
	Agricultural household = 0	2,571	0.597 (0.427)		2,723	2.427 (1.580)	
Marital status	married = 1	3,186	0.658 (0.402)	−1.68	3,311	2.719 (1.644)	2.68**
	Single = 0	1,091	0.633 (0.436)		1,148	2.868 (1.548)	
Education level	Higher education = 1	867	0.950 (0.128)	−25.71***	867	3.374 (1.206)	−12.74***
	Elementary education = 0	3,401	0.576 (0.423)		3,583	2.606 (1.672)	
Urban/rural	Urban = 1	2,498	0.725 (0.378)	−14.23***	2,555	3.089 (1.567)	−16.31***
	Rural = 0	1,779	0.548 (0.432)		1,904	2.311 (1.586)	
Economic region	Western = 0	1,713	0.679 (0.407)	8.39***	1,759	3.028 (1.607)	42.87***
	Central = 1	1,455	0.646 (0.419)		1,531	2.629 (1.627)	
	Eastern = 2	1,109	0.615 (0.404)		1,169	2.518 (1.577)	

Significance levels are indicated as follows: *p < 0.05, **p < 0.01, ***p < 0.001. Values in each cell represent the mean, with standard deviations in parentheses. Group differences were assessed using independent-samples *t*-tests for two-group comparisons and one-way ANOVA with *F*-tests for three-group comparisons. The difference in digital lifestyle by marital status was not statistically significant (*t* = −1.68, *p* = 0.092). For economic regions, post hoc pairwise comparisons were conducted using Scheffé’s test, with detailed results presented in the main text.

With the exception of marital status, all grouping variables exhibited significant differences in digital lifestyle. In terms of household registration, women with non-agricultural hukou had a significantly higher mean digital lifestyle score (*M* = 0.734) compared to their agricultural hukou counterparts (*M* = 0.597; *t* = −10.67, *p* < 0.001). Regarding educational level, women with higher education demonstrated a substantially higher mean digital lifestyle score (*M* = 0.950) than those with basic education (*M* = 0.576; *t* = −25.71, *p* < 0.001), representing the most pronounced disparity among all grouping variables. With respect to urban–rural classification, women residing in urban areas (neighborhood committee jurisdictions) had a significantly higher mean score (*M* = 0.725) than their rural counterparts (village committee jurisdictions; *M* = 0.548; *t* = −14.23, *p* < 0.001). For marital status, the difference between married women (*M* = 0.658) and unmarried women (M = 0.633) did not reach statistical significance (*t* = −1.68, *p* = 0.092). In terms of economic region, the mean digital lifestyle score exhibited a clear gradient pattern, with the eastern region having the highest score (*M* = 0.679), followed by the central region (*M* = 0.646) and the western region (*M* = 0.615). The overall difference across the three regions was statistically significant (*F* = 8.39, *p* < 0.001). *Post hoc* comparisons using Scheffe’s test revealed a significant difference between the western and eastern regions (*p* < 0.001), whereas the differences between the central and western regions (*p* = 0.166) and between the central and eastern regions (*p* = 0.078) were not statistically significant.

All grouping variables showed statistically significant differences in exercise frequency. In terms of household registration, women with non-agricultural hukou had a significantly higher mean exercise frequency (*M* = 3.269) than those with agricultural hukou (*M* = 2.427; *t* = −17.36, *p* < 0.001). Regarding marital status, unmarried women reported significantly higher exercise frequency (*M* = 2.868) compared to married women (*M* = 2.719; *t* = 2.68, *p* = 0.007), a pattern that contrasts with the non-significant marital status difference observed for digital lifestyle. With respect to educational level, women with higher education had a significantly higher mean exercise frequency (*M* = 3.374) than those with basic education (*M* = 2.606; *t* = −12.74, *p* < 0.001). In terms of urban–rural classification, urban women reported significantly higher exercise frequency (*M* = 3.089) than rural women (*M* = 2.311; *t* = −16.31, *p* < 0.001). Regarding economic region, exercise frequency also exhibited a significant gradient distribution, with the eastern region having the highest mean (M = 3.028), followed by the central (*M* = 2.629) and western (*M* = 2.518) regions. The overall difference across the three regions was statistically significant (*F* = 42.87, *p* < 0.001). Scheffe’s *post hoc* tests indicated that the differences between the eastern and central regions (*p* < 0.001) and between the eastern and western regions (*p* < 0.001) were significant, whereas the difference between the central and western regions was not (*p* = 0.203).

Overall, the distributional patterns of digital lifestyle and exercise frequency across different socio-demographic groups are largely consistent: women who are non-agricultural hukou holders, more educated, residing in urban areas, and living in the eastern region are positioned at a relative advantage in terms of both digital lifestyle engagement and exercise participation. These preliminary findings provide an empirical foundation for further examining the association between digital lifestyle and women’s exercise frequency, as well as its potential heterogeneity. Furthermore, they suggest that the differential effects posited in Hypothesis 2 warrant rigorous testing.

#### 4.2.2 Baseline regression

Table 4 presents the multiple linear regression results examining the association between digital lifestyle and women’s exercise frequency. A stepwise inclusion strategy of control variables was adopted, with a total of four models estimated to test the robustness of the relationship between digital lifestyle and women’s exercise frequency.

**Table 4:** Baseline regression of digital lifestyle on women’s exercise frequency.

Variables	Model 1 (*N* = 4,459)	Model 2 (*N* = 4,398)	Model 3 (*N* = 3,127)	Model 4 (*N* = 3,127)
Digital lifestyle	0.746*** (0.059)	0.702*** (0.080)	0.635*** (0.088)	0.568*** (0.088)
Age		0.008*** (0.002)	0.009*** (0.002)	0.008*** (0.002)
Household type		0.618*** (0.053)	0.585*** (0.059)	0.375*** (0.067)
Marital status		−0.048 (0.057)	−0.050 (0.063)	−0.033 (0.062)
Education level		0.425*** (0.073)	0.365*** (0.080)	0.318*** (0.080)
Social class			0.036** (0.017)	0.035** (0.017)
Economic status			−0.055 (0.038)	−0.051 (0.037)
Personal income			0.017*** (0.006)	0.015** (0.006)
Household economic status			0.046 (0.039)	0.049 (0.039)
Urban/rural				0.378*** (0.064)
Economic region				0.076**(0.033)
Constant	2.306^***^ (0.046)	1.613^***^ (0.145)	1.489^***^ (0.286)	1.343^***^ (0.286)
Observations	4,273	4,217	3,537	3,537
*R* ^2^	0.036	0.093	0.105	0.117

Standard errors are reported in parentheses. Sample sizes vary across models due to differences in the sets of control variables and missing data. All models were estimated using complete cases (i.e., listwise deletion of missing observations). Significance levels are indicated as follows: *p < 0.05, **p < 0.01, ***p < 0.001.

Model 1 included only the core explanatory variable (digital lifestyle) without any control variables. The results showed that the regression coefficient for digital lifestyle was 0.746 (*p* < 0.001), indicating a significant positive association between digital lifestyle and women’s exercise frequency. Specifically, for each one-unit increase in the Digital Lifestyle Index, women’s exercise frequency was expected to increase by 0.746 units.

Model 2 introduced individual-level characteristics, including age, household registration (hukou), marital status, and educational level, building upon Model 1. The results revealed that the coefficient for digital lifestyle decreased slightly from 0.746 to 0.702 (*p* < 0.001), suggesting that the core association was not substantially confounded by these individual-level characteristics. Among the newly added control variables, age (*β* = 0.008, *p* < 0.001) and hukou (*β* = 0.618, *p* < 0.001) were both significantly and positively associated with exercise frequency, indicating that older women and those with non-agricultural hukou reported higher exercise frequency. Educational level (*β* = 0.425, *p* < 0.001) was also significantly and positively associated with exercise frequency, with women holding higher education degrees exercising more frequently than those with basic education. The coefficient for marital status was negative but not statistically significant (*β* = −0.048, *p* > 0.05), suggesting no significant statistical association between marital status and women’s exercise frequency.

Model 3 further incorporated socio-economic characteristics, including social class, economic status, personal income, and household economic status, building upon Model 2. The results demonstrated that the coefficient for digital lifestyle further decreased to 0.635 but remained highly significant (*p* < 0.001), confirming that the positive association between digital lifestyle and exercise frequency remained robust even after controlling for individual-level and socio-economic factors. Among the socio-economic variables, social class (*β* = 0.036, *p* < 0.05) and personal income (*β* = 0.017, *p* < 0.01) were significantly and positively associated with exercise frequency, indicating that women with higher subjective social class and higher personal income exercised more frequently. The coefficients for economic status (*β* = −0.055, *p* > 0.05) and household economic status (*β* = 0.046, *p* > 0.05) did not reach statistical significance. It is worth noting that the sample size of Model 3 decreased by 680 observations compared to Model 2, which was largely attributable to missing values in variables such as social class, economic status, personal income, and household economic status.

Model 4, the most comprehensive specification, added two regional characteristics—urban–rural classification and economic region—to Model 3. The results showed that the regression coefficient for digital lifestyle was 0.568 (*p* < 0.001). Although somewhat attenuated compared to the previous three models, it remained highly significant. This finding indicates that even after simultaneously controlling for individual-level, socio-economic, and regional characteristics, digital lifestyle remained significantly and positively associated with women’s exercise frequency, thereby supporting Hypothesis 1. Among the regional characteristics, both urban–rural classification (*β* = 0.378, *p* < 0.001) and economic region (*β* = 0.076, *p* < 0.05) were significantly and positively associated with exercise frequency, indicating that urban women exercised more frequently than their rural counterparts, and women in the eastern region exercised more frequently than those in the central and western regions.

Across Models 1 through 4, with the gradual inclusion of additional control variables, the adjusted *R*^2^ values increased progressively from 0.036 to 0.117, suggesting that the included control variables explained a meaningful proportion of the variance in women’s exercise frequency. The *F*-statistics for all models were significant at the *p* < 0.001 level, indicating good overall model fit. The core explanatory variable, digital lifestyle, remained positively significant (*p* < 0.001) across all models, with its coefficient varying consistently and smoothly (from 0.746 to 0.568), without substantial fluctuations or sign reversals following the introduction of control variables. This further confirms the robustness of the positive association between digital lifestyle and women’s exercise frequency.

Taken together, the regression results from all four models support Hypothesis 1: there is a significant positive statistical association between digital lifestyle and women’s exercise frequency, such that women with higher levels of digital lifestyle engagement tend to exhibit higher exercise frequency.

#### 4.2.3 Robustness checks

To test the robustness of the baseline results, we conducted a series of robustness checks from three perspectives (Table 5).

**Table 5:** Robustness checks.

Variables	Model 5	Model 6	Model 7	Model 8	Model 9	Model 10
	Physical exercise participation	Physical exercise participation	Physical exercise participation	Physical exercise participation	Physical exercise participation	Frequency of moderate-to-vigorous physical activity
Digital lifestyle	0.515*** (0.155)					0.364** (0.158)
Frequency of internet use in the past year		0.123*** (0.021)				
Frequency of internet use during leisure time			0.151*** (0.019)			
Whether the internet is the primary source of information				0.252*** (0.070)		
Whether the respondent has used the internet in the past 6 months					0.430*** (0.071)	
Age	0.005 (0.004)	0.005** (0.002)	0.008*** (0.002)	0.003 (0.002)	0.005** (0.002)	0.003 (0.004)
Household type	0.337*** (0.117)	0.391*** (0.066)	0.374*** (0.066)	0.411*** (0.067)	0.397*** (0.066)	0.184 (0.118)
Marital status	0.022 (0.109)	−0.003 (0.061)	−0.031 (0.061)	0.006 (0.062)	−0.009 (0.061)	0.265** (0.113)
Education level	0.278** (0.139)	0.302*** (0.080)	0.320*** (0.079)	0.309*** (0.080)	0.333*** (0.080)	0.248* (0.142)
Social class	0.041 (0.031)	0.038** (0.017)	0.040** (0.017)	0.031* (0.017)	0.039** (0.017)	−0.003 (0.030)
Economic status	−0.048 (0.067)	−0.056 (0.037)	−0.056 (0.037)	−0.055 (0.038)	−0.056 (0.037)	−0.092 (0.065)
Personal income	0.025** (0.011)	0.016*** (0.006)	0.014** (0.006)	0.019*** (0.006)	0.015** (0.006)	−0.002 (0.011)
Household economic status	−0.010 (0.069)	0.036 (0.038)	0.033 (0.038)	0.059 (0.039)	0.037 (0.038)	0.124* (0.072)
Urban/rural	0.335*** (0.113)	0.371*** (0.063)	0.368*** (0.063)	0.404*** (0.064)	0.381*** (0.063)	0.034 (0.112)
Economic zone	0.128** (0.059)	0.086*** (0.033)	0.072** (0.032)	0.088*** (0.033)	0.092*** (0.032)	0.030 (0.058)
Health condition	0.035 (0.047)					
Body mass index	0.009 (0.013)					
Employment status	−0.123 (0.103)					
Mobile phone ownership	−0.221 (0.201)					
Local fitness infrastructure	0.243*** (0.044)					
Constant	0.481 (0.627)	1.433*** (0.281)	1.222*** (0.280)	1.705*** (0.282)	1.530*** (0.275)	2.038*** (0.509)
Observations	1,171	3,672	3,664	3,550	3,669	1,189
*R* ^2^	0.141	0.119	0.126	0.110	0.120	0.038

The variables “local fitness infrastructure” and “moderate-to-vigorous physical activity” were drawn from the EASS health module of CGSS 2021, which used a random one-third sub-sample design. The smaller and similar sample sizes in Models 5 and 10 are thus by design, not due to missing data. Significance levels are indicated as follows: *p < 0.05, **p < 0.01, ***p < 0.001. Standard errors are reported in parentheses.

First, additional control variables. Building upon the baseline model, we incorporated five additional variables: self-rated health status, body mass index (BMI), employment status, mobile phone ownership, and perceived suitability of the residential environment for physical exercise. The results (Model 5) showed that the regression coefficient for digital lifestyle was 0.515 (*p* < 0.001), which remained consistent in direction and significance with the baseline estimate (*β* = 0.568), suggesting that the findings are not severely biased by omitted variables.

Second, alternative measurement of the independent variable. We substituted the composite Digital Lifestyle Index with each of its four constituent indicators as the core explanatory variable in separate regressions. The results (Models 6–9) demonstrated that all four indicators yielded positive and significant coefficients (*p* < 0.001), ruling out the possibility that the observed findings are driven by the specific construction method of the composite index.

Third, alternative measurement of the dependent variable. We replaced the dependent variable with “frequency of moderate-to-vigorous physical activity,” which was measured by the question asking how often respondents engaged in physical activity that lasted at least 20 min and caused sweating or rapid breathing. The results (Model 10) indicated that the coefficient for digital lifestyle remained positive and significant (*β* = 0.364, *p* < 0.05).

In summary, after (1) adding additional control variables, (2) employing alternative measures of the independent variable, and (3) employing an alternative measure of the dependent variable, the core findings remained robust. These results demonstrate that the baseline estimates are reliable.

#### 4.2.4 Heterogeneity analysis

To further examine whether the association between digital lifestyle and women’s exercise frequency varies across different socio-demographic groups (H2), we employed the Chow test approach by estimating multiple regression models that included interaction terms between each grouping variable and the digital lifestyle index. All models incorporated the same set of control variables as the baseline model (Model 4), including age, household registration (hukou), marital status, educational level, social class, economic status, personal income, household economic status, urban–rural classification, and economic region. The reference categories for each grouping variable were as follows: age group (reference = women under 55 years and men under 60 years), hukou (reference = agricultural), marital status (reference = not married), educational level (reference = basic education or below), urban–rural classification (reference = rural), and economic region (reference = western, treated as a continuous variable: 1 = western, 2 = central, 3 = eastern). The results are presented in Table 5.

**Figure 1:**
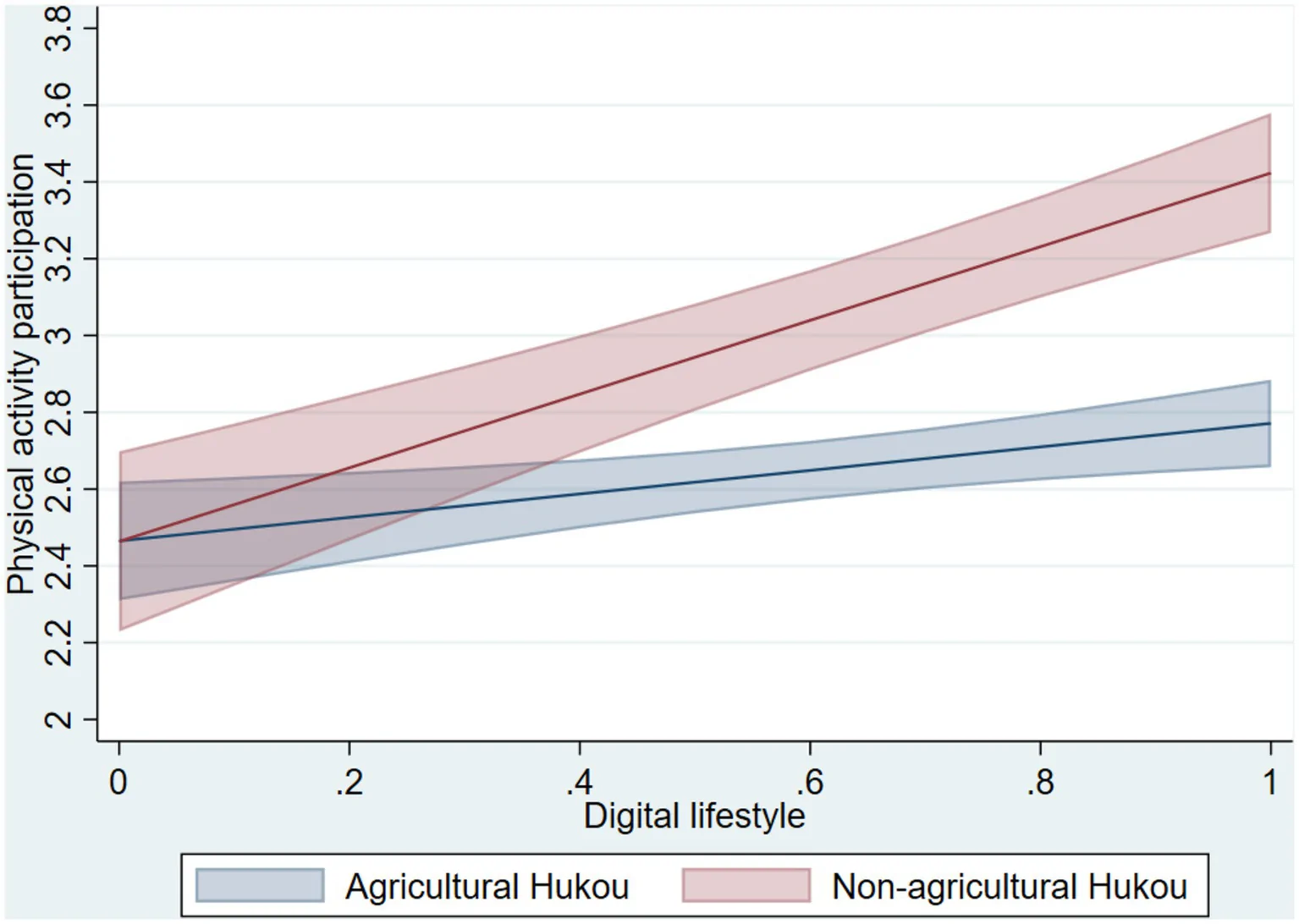
Heterogeneity by household registration.

First, the moderating effect of household registration. The interaction term between hukou and digital lifestyle yielded a coefficient of 0.654 (*p* < 0.001), which was statistically significant, indicating that the positive association between digital lifestyle and exercise frequency was significantly stronger among women with non-agricultural hukou than among their agricultural hukou counterparts. Figure 1 presents the marginal effects plot, which visually illustrates this pattern: as the digital lifestyle index increased, the predicted exercise frequency for non-agricultural hukou women exhibited a steep upward trajectory (slope ≈ 2.0), whereas the increase for agricultural hukou women was relatively moderate (slope ≈ 1.0), with the two lines diverging in a scissor-like pattern. This disparity may be attributable to the structural advantages enjoyed by non-agricultural hukou women in terms of digital infrastructure access (e.g., broadband internet, smart devices), digital literacy (information retrieval and evaluation capabilities), and health resource accessibility (e.g., affordability of online fitness platforms) (van Scheerder et al., 2019). Drawing on Field Theory, the virtual fitness field constructed by digital technologies is not value-neutral; rather, its entry and utilization are inherently constrained by the volume and composition of capital—particularly cultural and economic capital—that individuals possess in the traditional social space. Women with non-agricultural hukou, by virtue of their higher educational attainment and income levels, are better positioned to effectively translate digital lifestyle engagement into actual physical exercise behavior (Bourdieu, 1988).

**Figure 2:**
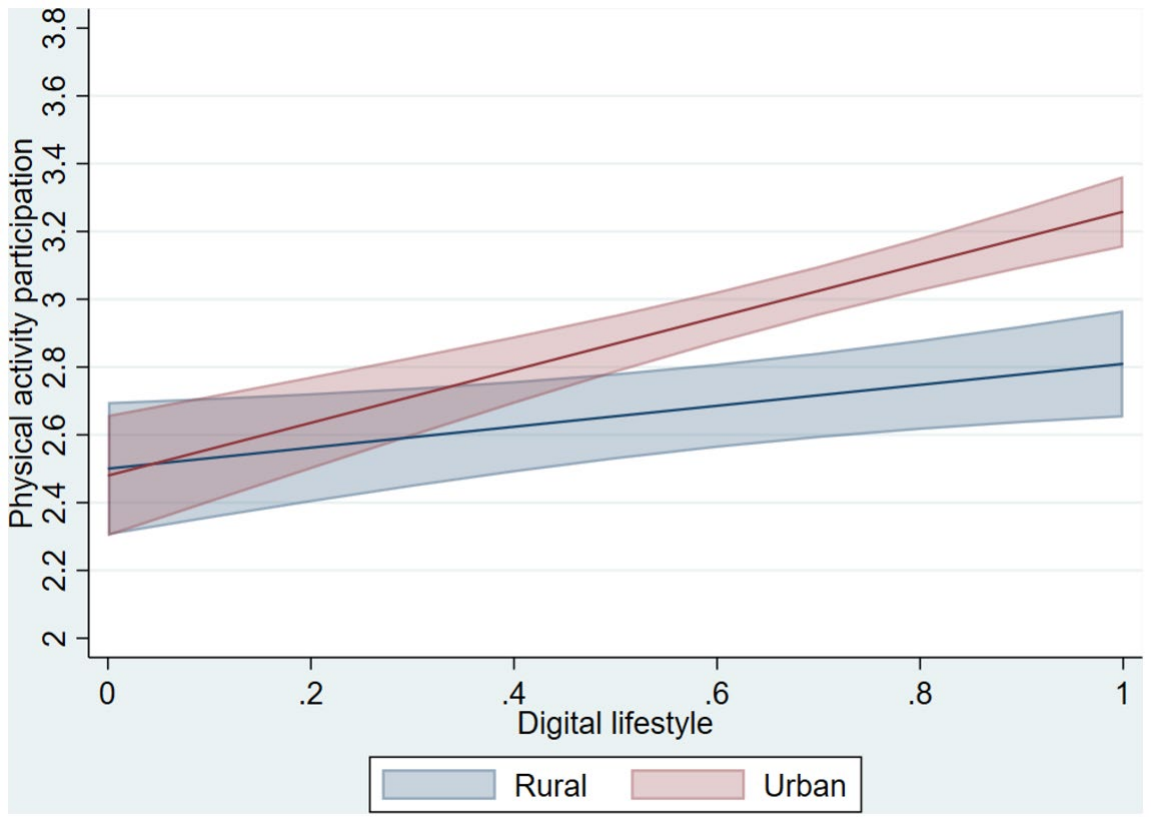
Heterogeneity by urban–rural classification.

Second, the moderating effect of urban–rural classification. The interaction term between urban–rural classification and digital lifestyle yielded a coefficient of 0.470 (*p* < 0.01), which was statistically significant, indicating that the positive association between digital lifestyle and exercise frequency was significantly stronger among urban women than among their rural counterparts. Figure 2 similarly illustrates this pattern: as digital lifestyle engagement increased, the predicted exercise frequency for urban women exhibited a clear upward trend, whereas the increase for rural women remained relatively limited. This disparity is closely related to the long-standing “digital divide” between urban and rural areas—rural regions continue to lag behind urban areas in terms of internet access quality (e.g., network bandwidth, mobile signal coverage), the availability of digital fitness resources (e.g., the penetration and promotion of online exercise programs), and the depth of digital health information dissemination (Yong, 2021). From the perspective of Field Theory, the social space inhabited by rural women lacks the supportive conditions necessary for the effective functioning of the digital fitness field—such as a stable internet environment, adequate physical space for home-based exercise, and a community-level fitness culture—such that even when rural women have access to digital technologies, they may struggle to translate this access into sustained physical exercise practice (Hargittai, 2018).

Third, grouping variables with non-significant moderating effects. The interaction terms for age group (coefficient = 0.194, *p* > 0.05), marital status (coefficient = −0.342, *p* > 0.05), educational level (coefficient = −0.223, *p* > 0.05), and economic region (coefficient = 0.212, *p* > 0.05) did not reach statistical significance. This indicates that the positive association between digital lifestyle and exercise frequency remained consistent across women of different age groups, marital statuses, educational levels, and economic regions, with no significant between-group heterogeneity detected. Notably, the non-significant moderating effect of educational level deserves particular attention—although women with higher education exhibited significantly higher levels of both digital lifestyle engagement and exercise frequency (see Table 2), the “facilitative effect” of digitalization on exercise behavior did not differ significantly between women with different educational backgrounds. This finding carries positive policy implications: the potential of digital technologies to promote physical exercise appears to hold across educational strata, suggesting that the proliferation of digital fitness resources may, to some extent, circumvent the traditional constraints that educational stratification imposes on health behaviors (Cotter et al., 2020).

Taken together, the heterogeneity analysis results (see Table 6) indicate that Hypothesis 2 was partially supported: the association between digital lifestyle and women’s exercise frequency exhibited significant heterogeneity by hukou and urban–rural classification, but remained consistent across age, marital status, educational level, and economic region. The dual disparities rooted in hukou and urban–rural classification suggest that the institutional barriers constructed by China’s hukou system and urban–rural dual structure continue to function as structural constraints on women’s exercise participation, even in an era of deep digital penetration (Henderson et al., 1989).

**Table 6:** Heterogeneity analysis.

Variables	Age group	Household registration	Marital status	Educational level	Urban–rural classification	Economic region
*N*	3,537	3,537	3,537	3,537	3,537	3,537
Digital lifestyle	0.413** (0.158)	0.306** (0.111)	0.887*** (0.199)	0.545*** (0.09)	0.309** (0.128)	0.303 (0.158)
Digital lifestyle × Age group	0.194 (0.186)					
Digital lifestyle × Hukou		0.654*** (0.183)				
Digital lifestyle × Marital status			−0.342 (0.222)			
Digital lifestyle × Educational level				−0.223 (0.500)		
Digital lifestyle × urban–rural classification					0.470** (0.177)	
Digital lifestyle × economic region						0.212 (0.111)
Constant	1.628*** (0.357)	1.793*** (0.369)	0.757 (0.571)	1.177*** (0.315)	1.267*** (0.436)	1.644*** (0.484)
*F*	24.11***	23.82***	24.46***	24.29***	23.93***	23.69***
*R* ^2^	0.126	0.125	0.128	0.127	0.125	0.124

All models include the same controls as Model 4 (age, hukou, marital status, education, social class, economic status, personal income, household economic status, urban–rural, economic region). Main effects of grouping variables and their interactions with other controls are not fully reported. Reference groups: age (<55 women/<60 men), hukou (agricultural), marital status (not married), education (basic or below), urban–rural (rural), economic region (western). Economic region is treated as a continuous variable (0 = western, 1 = central, 2 = eastern). Significance levels are indicated as follows: *p < 0.05, **p < 0.01, ***p < 0.001. Standard errors are reported in parentheses.

## 5 Study 2

### 5.1 Methods

#### 5.1.1 Study design and participants

This qualitative component adopts a constructivist grounded theory approach (Charmaz, 2014), aiming to develop a theoretical model that explains the underlying mechanisms through which digital lifestyles influence women’s exercise behaviors. This approach is particularly suited for exploring social processes where existing theoretical explanations are limited, as established in Study 1.

Participants were recruited using a purposive sampling strategy to ensure diversity in age, occupation, and living context, which are key factors identified in the quantitative phase. Initial participants were recruited through community centers and online fitness groups. Subsequent participants were selected using theoretical sampling, a core tenet of grounded theory, where data collection and analysis occur iteratively. This means that as initial codes and categories emerged (e.g., the importance of “online communities”), we actively sought out new participants who could further elaborate on these specific categories (e.g., women heavily involved in online fitness groups), until theoretical saturation was achieved (Juliet and Corbin, 2015).

A total of 12 women participated in this study. Their demographic information is presented in Table 6. The sample includes a mix of full-time mothers, professionals, and students, with ages ranging from their 20s–40s, residing in both urban and peri-urban areas in China. This diversity allows for a comprehensive exploration of the phenomenon across different life contexts (Table 6).

#### 5.1.2 Data collection

Data were collected through semi-structured, in-depth interviews conducted between [Insert Month, Year] and [Insert Month, Year]. All interviews were conducted in Mandarin Chinese by the first and corresponding authors, who are trained in qualitative research methods. The interviews took place either face-to-face in a private room at a community center or via secure video conferencing platforms, based on the participant’s preference and availability. Each interview lasted between 20 and 60 min (mean duration = 35 min).

An interview guide was developed based on the literature review and the findings of Study 1. The guide was used flexibly to allow for emergent topics. Key questions included:

“Can you describe a typical day and how you incorporate (or don’t incorporate) physical activity?”

“How, if at all, do you use digital devices (like smartphones, apps, or social media) in relation to your health and exercise?”

“Can you tell me about a time when digital technology motivated you to exercise, or perhaps, discouraged you?

“How do the online fitness communities you participate in (if any) affect your exercise routine?”

This part of the study was approved by the Medical Ethics Committee of the School of Medicine, Shenzhen University (Approval Number: PN-202500030). Informed consent was obtained from all participants before the interviews. Participants were informed about the purpose of the study, their right to withdraw at any time, and that their identities would be kept confidential. All interviews were audio-recorded with the participants’ explicit consent. The recordings were then transcribed verbatim in Chinese by a professional transcription service and subsequently checked for accuracy by the research team. Participants were offered the opportunity to review their transcripts to ensure their experiences were accurately captured (member checking) (see Table 7).

**Table 7:** Interview participant information.

Index	Name	Gender	Age (range)	Occupation
1	WZY	W	Born after 1995	Administrative management
2	CLJ	W	Born after 2000	Administrative management
3	LXR	W	Born after 1990	Teacher
4	ZMY	W	Born after 1990	Unemployed
5	LHX	W	Born after 1985	Professor
6	ZYN	W	Born after 1990	Salesperson
7	YST	W	Born after 1990	Civil servant
8	HQL	W	Born after 1995	Full-time mother
9	XRX	W	Born after 1995	Bank employee
10	SWY	W	Born after 2000	No fixed occupation
11	GJH	W	Born after 1985	Self-employed
12	ZTF	W	Born after 1980	Full-time mother

#### 5.1.3 Data analysis

The analysis was conducted in Chinese, the original language of the data, to preserve the nuances of participants’ expressions. Key concepts and themes were translated into English during the writing phase. We followed a systematic coding process:

##### 5.1.3.1 Initial (open) coding

The transcripts were line-by-line coded by two researchers independently (Author 1 and Author 2). This stage yielded 437 initial codes (e.g., “time flexibility,” “peer pressure to exercise,” “fear of being judged online”).

##### 5.1.3.2 Focused (axial) coding

The research team (all authors) met regularly to compare their independent codes. Through an iterative process of discussion and consensus, we grouped the initial codes by conceptual similarity, merging, revising, or discarding overlapping or irrelevant codes. This process resulted in 143 focused codes.

##### 5.1.3.3 Theoretical (selective) coding

The focused codes were then categorized into more abstract analytical dimensions. Through constant comparison between codes, categories, and the emerging theoretical framework, we synthesized 13 analytical dimensions (e.g., “Construction of a Digital Field,” “Acquisition of Cultural Capital,” “Internalization of Digital Habits”).

##### 5.1.3.4 Theory building

These dimensions were further integrated to identify 10 key factors that, in conjunction with Bourdieu’s theoretical concepts, formed the core components of our final model: Field, Capital, and Habitus.

#### 5.1.4 Rigor and trustworthiness

To ensure the rigor and credibility of our qualitative findings, we employed several strategies recommended by Lincoln and Guba (1988):

##### 5.1.4.1 Credibility

We used triangulation of data sources (by including participants with diverse backgrounds) and investigator triangulation (by having multiple coders). We also conducted member checking by returning to a subset of participants (*n* = 3) with a summary of our preliminary findings to ensure they resonated with their experiences.

##### 5.1.4.2 Transferability

Thick description of participants’ backgrounds and the research context is provided to allow readers to assess the applicability of our findings to other settings.

##### 5.1.4.3 Dependability and confirmability

A detailed audit trail of all research decisions, coding steps, and memos was maintained throughout the research process. The use of multiple coders and the consensus process helped to minimize individual researcher bias. Theoretical saturation was deemed to have been achieved when no new properties, dimensions, or relationships emerged during the analysis of the final three interviews, and the developed categories were well-integrated and robust (Guest et al., 2006).

### 5.2 Findings

#### 5.2.1 Field theory: the construction of a digital sports environment

**Figure 3:**
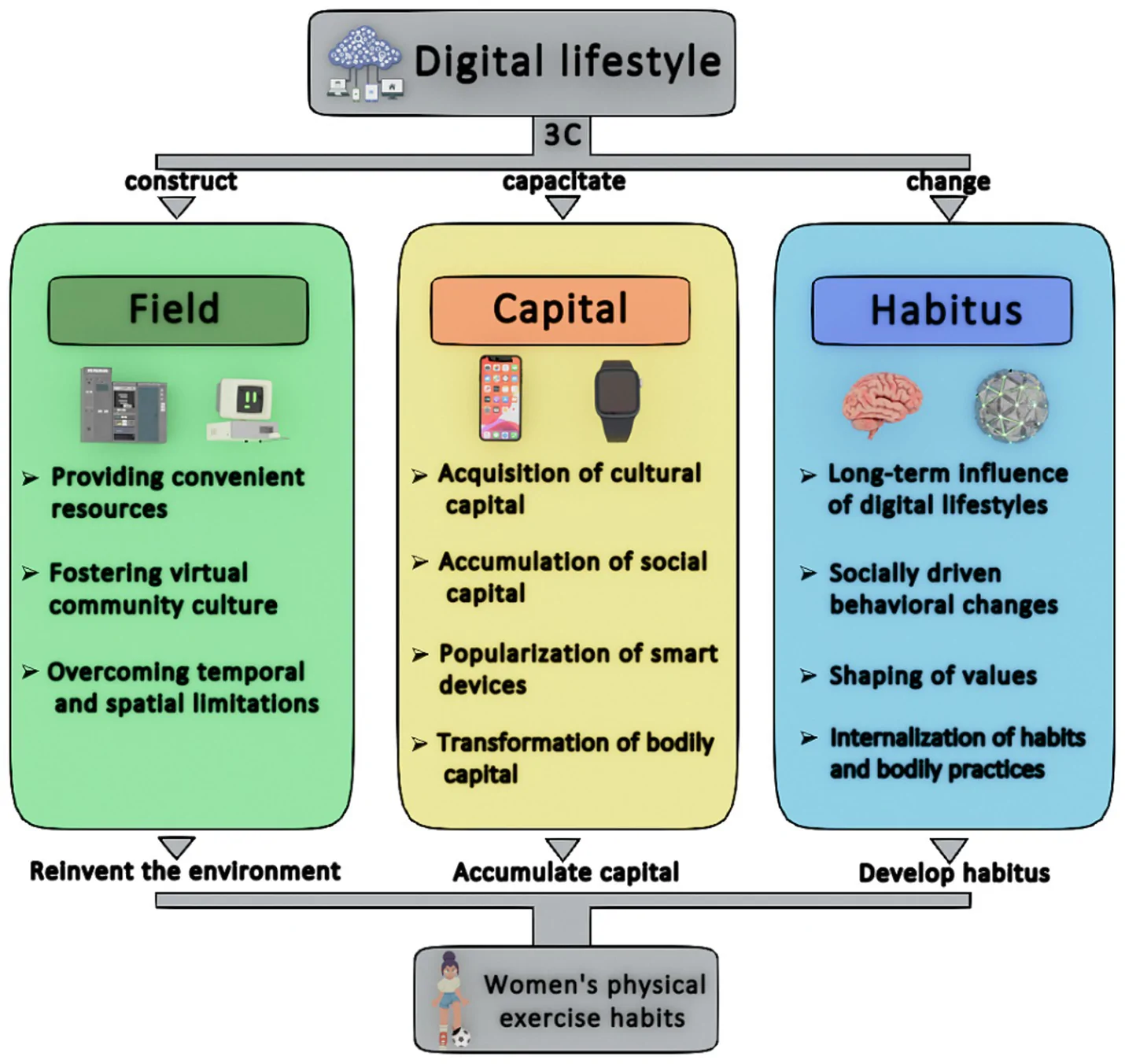
Pathway of how digital lifestyles influence women’s exercise habits.

The term *field* refers to a specific social space where individuals engage in activities and interactions. Under the influence of digital lifestyles, physical exercise habits have expanded from traditional fields (such as gyms and parks) to digital fields (such as online fitness platforms and social media fitness communities). These digital fields integrate information flow, technological tools, and social interactions, reshaping the environment in which women participate in physical exercise (see Figure 3).

Digital fields provide easily accessible exercise resources, such as online courses, live-streamed fitness sessions, and personalized workout plans. As the interviewee HJ shared: “*Digital platforms provide us with many fitness resources. After giving birth, I mainly followed app-based courses for postpartum recovery*.”

Moreover, social media fitness communities foster a virtual group culture that encourages women to engage in exercise more actively through user-generated content and interaction. A good example is what the interviewee WZY stated, “*We have a group chat where we share photos or videos of our workouts every day. This really motivates me. At one point, many of us were following Liu Genghong’s workout routines together every day.*”

At the same time, digital fields overcome time and space constraints, providing flexible workout opportunities—particularly for women who are busy with work or family responsibilities. Through this reconstruction of the physical exercise field, digital lifestyles enable women to engage in and adapt to new modes of sports participation.

#### 5.2.2 Capital: how digital tools empower women’s exercise capital

Digital Tools refer to applications or devices that leverage digital technology or software to support users in completing specific tasks or solving particular problems. These tools typically incorporate one or more functions designed to enhance efficiency, improve user experience, and optimize performance in areas such as productivity, health, and learning.

##### 5.2.2.1 Acquisition of cultural capital

Cultural capital refers to the knowledge, skills, and habits that individuals acquire through education, experience, and cultural accumulation (Lareau and Weininger, 2003). In the digital fitness field, women gain knowledge and skills related to exercise and health by participating in online courses and watching fitness videos. These competencies help them establish a healthy and active image in society, earning recognition and support from others. As the interviewee LXR said, “*By following fitness influencers on Xiaohongshu, I have learned a lot about exercise techniques and dietary methods. I feel like I understand better how to maintain my health, and I’m increasingly eager to share these experiences with my friends*.”

##### 5.2.2.2 Accumulation of social capital

Within social media environments, women enhance interactions and connections with others through fitness check-ins and workout progress sharing, receiving social support and encouragement. This social interaction helps them stay motivated to exercise and fosters a sense of group identity. With social capital as a support system, women are more likely to sustain and integrate exercise routines into their daily lives. For example, an interviewee, HQL, noted, “*I joined a fitness check-in group on Xiaohongshu, where we encourage and share our progress with each other every day. This has helped me stay consistent. Seeing everyone making progress together gives me even more motivation.*”

##### 5.2.2.3 Popularization of smart devices

The widespread adoption of smart devices and fitness applications has enabled women to engage in personalized workouts at home. However, the accessibility of economic capital influences women’s engagement in digital fitness spaces. Some women, due to better financial conditions, can afford high-end smart fitness equipment, while others rely on free fitness apps. This economic disparity affects their position and participation in the digital fitness field. A low-income interviewee, ZMY, remarked, “*I can’t afford expensive fitness equipment, but I use free apps to learn yoga and strength training. Although my equipment isn’t as professional, it still helps me maintain my exercise routine.*”

##### 5.2.2.4 Transformation of bodily capital

Digital tools, such as smartwatches and fitness trackers, allow women to monitor their physical condition in real-time, helping them adjust their workout plans and dietary habits. Through these digital devices, women can better manage their physical capital, fostering self-regulation and self-awareness. This process contributes to the redefinition and enhancement of their health and body image. As the interviewee ZYN shared, “*Since I started using a smartwatch, I can track my daily steps and calories burned. This motivates me to exercise more, and I feel like I am taking control of my health.*”

#### 5.2.3 Habitus: transformations in women’s exercise perceptions and practices

##### 5.2.3.1 Long-term influence of digital lifestyles

Women are gradually becoming accustomed to using fitness apps and smart devices to record and manage their health. This digitalized habit is increasingly internalized as part of their daily routines. For example, ZMY said, “*I check my WeChat step count every day. Seeing how my steps change each day motivates me to walk or run more, and over time, I have developed a habit of daily exercise.*” Likewise, the interviewee YST stated, “*I use a fitness app to track my diet and exercise data, and I’ve gotten used to monitoring my weekly progress. The digital feedback makes exercise feel more structured rather than just an effort without clear results.*” This dependence on digital tools is not only a method of health management but also a socialization process. Through long-term data feedback, women unconsciously develop a data-driven approach to maintaining their health. Several interviewees mentioned that instant feedback and visualized progress tracking provided by digital devices and apps help them maintain a consistent health management routine.

##### 5.2.3.2 Socially driven behavioral changes

Social media fitness trends and challenge activities, such as the “21-day Exercise Challenge” or fitness check-ins in WeChat Moments, encourage women to engage in physical exercise and gradually transform their workout habits. Through social interactions, women not only gain emotional support but also develop a stronger sense of belonging to the fitness culture, reinforcing their commitment to exercise.

For example, interviewee HQL shared, “*I joined an online fitness check-in challenge. Initially, I just wanted to try it out, but I didn’t expect to receive so much encouragement from the group every day. That’s what kept me going. Every time I saw others posting their workout photos or videos, I felt compelled to join in. Eventually, it became a habit.*”

This kind of social interaction mechanism can sustain people’s workout behaviors and internalize exercise habits through group recognition and encouragement.

##### 5.2.3.3 Shaping of values

With the internalization of digital lifestyles, women’s understanding and approach to health have evolved. Influenced by social media and digital platforms, their health perceptions have become more holistic and personalized, moving beyond traditional emphasis on appearance and weight loss. Interviewee XRX noted, “*I used to think fitness was only for weight loss, but after following health influencers and taking online courses, I realized that exercise benefits not only weight management but also mental health, cardiovascular health, and immunity. Now, I see exercise as part of my life—even something as simple as yoga makes me feel like I’m taking care of both my body and mind.*” This perspective shift illustrates the profound impact of digital fitness platforms on women’s health perceptions. Similarly, another interviewee, SWY, added, “*Through various fitness communities, I have not only learned exercise techniques but also gained knowledge about nutrition and mental health. Over time, fitness has become less about body shape and more about maintaining overall physical and mental well-being.*”

##### 5.2.3.4 Internalization of habits and bodily practices

Through repetitive engagement with digital fitness tools and social interactions, women internalize exercise habits as part of their embodied practices. With the support of smart devices and fitness platforms, they can manage their health more precisely, gradually developing a regular exercise routine. Interviewee GHJ shared her experience, “*Since I started using a smartwatch, I check my step count, calorie consumption, and heart rate every day. I’ve also begun monitoring my sleep quality. At first, I was just curious about the data, but over time, I realized that tracking these metrics helped me better understand my body and adjust my workout plans accordingly. These habits have now become part of my daily life, and I hardly need any reminders to keep up with them.*”

## 6 Discussion

### 6.1 Theoretical contribution of study 1

First, this study highlights how digital empowerment helps break traditional gender constraints in sports. Historically, sports have been perceived as a male-dominated domain, reflecting entrenched patriarchal structures and gender inequalities, where women are often marginalized due to gendered divisions of labor (Spaaij et al., 2015). Using large-scale empirical data, this study finds that digital lifestyles significantly and positively influence women’s exercise frequency, suggesting that digital technology can overcome traditional spatial, temporal, and institutional barriers, thereby providing women with greater access to sports participation. This finding underscores the potential of digitalization to facilitate gender empowerment in the sports field.

Besides, the empirical results support Feminist Sports Theory’s advocacy for equal participation. Liberal feminism argues that institutional reforms and equal resource distribution are essential for improving women’s status in sports. The findings reveal that women from different socioeconomic backgrounds (e.g., urban–rural residence, household type, education level) exhibit significant disparities in their ability to utilize digital resources for exercise participation. This aligns with feminist perspectives, which assert that even when women gain access to opportunities in a male-dominated social structure, unequal resource distribution continues to limit their full development (Roth and Basow, 2004). This study provides new empirical evidence on how digital tools can be leveraged to promote gender equality in sports.

Moreover, this study extends the application of Feminist Sports Theory beyond traditional sports domains. Some scholars argue that Feminist Sports Theory not only addresses inequalities within traditional sports settings but also advocates for reconstructing new spaces for practice, enabling women to express themselves and achieve bodily emancipation across broader domains (Markula, 2003). Through quantitative analysis, this study demonstrates how digital lifestyles expand the boundaries of sports participation, creating a “digital field” where women can engage in exercise at home, on online platforms, and in diverse virtual spaces. This expansion not only broadens women’s avenues for sports engagement but also provides theoretical support for redefining gender relations in sports, reinforcing the idea that digitalization can serve as a tool for restructuring traditional gender norms in physical activity.

### 6.2 Contribution of study 2

First, study 2 helps construct a theoretical framework for the digital sports field. Traditionally, a field is socially constructed through shared logics, serving as both the primary space for individuals to engage in social activities and a site for symbolic competition and strategic actions (Perreault and Bell, 2022). Through in-depth interviews and grounded theory analysis, this study reveals how digital lifestyles reshape the spatial and participatory dynamics of traditional sports engagement. Drawing on Bourdieu’s field theory, Study 2 conceptualizes digital platforms, online fitness communities, and smart devices as key elements in reconfiguring the sports field. With the rise of digital technology, sports participation is no longer confined to physical spaces such as gyms or outdoor venues; instead, it unfolds within a fluid, interactive, and technology-driven digital sports field. With the rise of digital technology, sports participation is no longer confined to physical spaces such as gyms or outdoor venues; instead, it unfolds within a fluid, interactive, and technology-driven digital sports field. This reconceptualization advances theoretical discussions on how technological innovation restructures existing social fields, particularly in the context of health and exercise behaviors.

Besides, this study uncovers the mechanism of sports habitus regeneration. Habitus, as a form of socialized subjectivity, is a dynamic and evolving system of dispositions that shape individual behaviors and choices (Gouanvic, 2005). According to field theory, habitus is both a product of and a contributor to the social field, constructed through capital distribution, institutional environments, and lived experiences (Martin, 2003). Study 2 reveals that the digital environment fundamentally alters how individuals access information and engage in physical activity, fostering a sports habitus distinct from traditional forms. This transformation exemplifies the reciprocal relationship within Bourdieu’s theoretical model, encapsulated in the equation: habitus × capital + field = practice. Within the digital sports field, digital capital, social capital, and cultural capital interact to reshape women’s exercise behaviors, demonstrating how technological affordances influence habitus formation and lifestyle choices.

Last, this study extends the application of field theory in the digital age. Field theory posits that fields are not static entities but are continuously shaped by technological, cultural, and structural transformations (Perreault and Bell, 2022). This study provides empirical evidence of how digitalization disrupts traditional spatial and temporal constraints, reconfigures social interactions among fitness participants, and reconstructs the conventional sports field. By integrating qualitative insights into digital fitness participation, Study 2 enriches the application of field theory in contemporary sports sociology, illustrating how capital, habitus, and individual agency intersect in the digital era. In doing so, this study extends Bourdieu’s theoretical framework to digitalized health and fitness behaviors, contributing to broader discussions on technology’s role in shaping modern social practices.

### 6.3 Possible solutions

The digital divide remains a significant barrier to rural women’s participation in physical exercise, as digital lifestyles have been shown to have a greater impact on urban women while rural women benefit less. To address this disparity, efforts should focus on expanding digital access in rural areas. Governments should improve internet infrastructure, increase network coverage, and provide subsidies for digital fitness devices, ensuring that rural women have equal access to online fitness resources. By narrowing this gap, more rural women can integrate digital tools into their exercise routines, fostering healthier lifestyles.

Social support networks play a crucial role in bridging the connection between digital usage and exercise participation. Strengthening these networks can further enhance women’s engagement in fitness activities. One approach is to develop online fitness communities through social media platforms and fitness apps, where women can share experiences, join fitness challenges, and cultivate a sense of belonging. Additionally, a hybrid “online + offline” fitness model can be implemented, combining virtual fitness programs with community-based exercise initiatives in both urban and rural areas. By facilitating social interactions and peer encouragement, these initiatives can help sustain women’s motivation to engage in physical activity.

To increase women’s participation in digital fitness, enterprises and governments should collaborate to develop women-specific fitness content. Leveraging data analysis and AI technology, personalized workout plans can be designed based on age, occupation, and health conditions, making exercise more tailored and effective. Furthermore, digital fitness platforms should offer specialized courses addressing women’s health needs, such as pregnancy fitness, postpartum recovery, and stress management, to enhance engagement and long-term adherence. By ensuring that fitness content is inclusive and responsive to women’s needs, digitalization can become a more powerful tool in promoting active and healthy lifestyles among women across diverse backgrounds.

### 6.4 Integrating quantitative findings with qualitative mechanisms: a mixed-methods perspective

This mixed-methods design allows us to move beyond a simple correlational finding to a nuanced understanding of how and for whom digitalization promotes exercise. The quantitative findings of Study 1 establish three key patterns: (1) a positive main effect of digital lifestyle on exercise frequency (H1), (2) a weaker effect for rural women compared to urban women, and (3) a stronger effect for more educated women. The qualitative findings from Study 2 provide a theoretical lens to interpret these patterns.

First, the overall positive effect (H1) can be explained by the mechanisms uncovered in Study 2. The qualitative data show that digitalization does not merely add an activity; it reconstructs the entire field of practice. It provides flexible access (breaking temporal/spatial constraints), builds social capital through online communities (providing motivation and accountability), and facilitates the acquisition of health knowledge (cultural capital). These mechanisms collectively explain the “why” behind the “what” of Study 1.

Second, the disparity between urban and rural women is illuminated by the qualitative analysis of capital. Rural interviewees in our sample frequently mentioned a lack of confidence and knowledge, signaling a lower level of embodied cultural capital. Urban women, in contrast, spoke about integrating diverse digital resources. This suggests that the same digital field does not offer equal opportunities; it provides a new “playing field,” but one where the capacity to play is still unevenly distributed. The digital divide, therefore, is not just about access to technology (as measured in CFPS), but also about access to the requisite social and cultural capital to effectively use that technology.

Third, education acted as a strong moderator in Study 1. The qualitative data provides a mechanism for this: educated women demonstrated a greater capacity to critically evaluate and curate digital fitness content. They were more likely to seek out evidence-based programs and set strategic goals, a process reflecting the conversion of cultural capital (formal education) into health capital. As LXR mentioned, “I don’t just follow any video; I look for instructors with credible credentials and a science-based approach.” This strategic engagement contrasts with less-educated participants, who, like ZMY, felt overwhelmed by the sheer volume of content and often defaulted to free, popular, but unstructured content. This shows how pre-existing cultural capital shapes the effectiveness of digital capital in producing health outcomes.

### 6.5 Limitations and further directions

Several limitations of this study should be acknowledged, which also point to directions for future research.

First, the empirical analysis relies exclusively on data from China, which limits the cross-national generalizability of our findings. Cultural, economic, and technological contexts vary considerably across countries, and the relationship between digital lifestyle and women’s exercise behavior observed in China may not hold in other settings. Future research could incorporate cross-national longitudinal datasets to examine whether the patterns identified here are context-specific or reflect broader global trends.

Second, while the qualitative component of this study provides an in-depth exploration of the mechanisms through which digital lifestyles influence women’s exercise habits, the analysis remains at an exploratory level. Due to the scope of the current study, we did not conduct exhaustive coding saturation, and some potentially relevant factors may not have been fully captured. Future research could expand the qualitative inquiry by including participants from diverse backgrounds—such as varying occupational roles, family structures, and geographic locations—to further refine and validate the field model of digital lifestyle influences on women’s exercise participation proposed in this study.

Third, the quantitative findings of this study are based on cross-sectional survey data, which precludes causal inference. Although we included a comprehensive set of control variables to minimize omitted variable bias, we cannot rule out the possibility of reverse causality—that is, women with higher exercise frequency may be more inclined to actively use digital tools, rather than digital lifestyles driving exercise participation. Moreover, unmeasured confounders—such as individual health consciousness, intrinsic exercise motivation, and peer influence—may also have contributed to the observed associations. Therefore, our results should be interpreted as evidence of statistical association rather than causal effects. Future research employing longitudinal designs or quasi-experimental approaches—such as instrumental variable estimation, difference-in-differences, or regression discontinuity designs—would be valuable for establishing the causal direction and magnitude of the relationship between digital lifestyle and women’s physical exercise.

## Data Availability

The original contributions presented in the study are included in the article/supplementary material, further inquiries can be directed to the corresponding author.

## References

[ref1] Andersen P. L. Bakken A. (2019). Social class differences in youths’ participation in organized sports: what are the mechanisms? Int. Rev. Sociol. Sport 54, 921–937. doi: 10.1177/1012690218764626

[ref2] Belabbes M. A. Ruthven I. Moshfeghi Y. Rasmussen Pennington D. (2023). Information overload: a concept analysis. J. Doc. 79, 144–159. doi: 10.1108/jd-06-2021-0118

[ref3] Bourdieu P. (1988). Homo Academicus. Stanford, California, USA: Stanford University Press.

[ref4] Carraça E. Encantado J. Battista F. Beaulieu K. Blundell J. Busetto L. . (2021). Effective behavior change techniques to promote physical activity in adults with overweight or obesity: a systematic review and meta-analysis. Obes. Rev. 22:e13258. doi: 10.1111/obr.13258 33949778 PMC8365685

[ref9001] Charmaz K. (2014). Constructing grounded theory (introducing qualitative methods series). Constr. grounded theory.

[ref9002] Cotter J. C. Wilson K. J. Mallonee L. F. (2020). Impact of HPV immunization training on dental hygiene students’ attitudes and confidence regarding HPV preventive education. Journal of Dental Education, 84, 88–93, . 31977100 10.21815/JDE.019.164

[ref5] De Santis K. Jahnel T. Sina E. Wienert J. Zeeb H. (2021). Digitization and health in Germany: cross-sectional nationwide survey. JMIR Public Health Surveill. 7:e32951. doi: 10.2196/32951, 34813493 PMC8612128

[ref6] Domin A. Spruijt-Metz D. Theisen D. Ouzzahra Y. Vögele C. (2021). Smartphone-based interventions for physical activity promotion: scoping review of the evidence over the last 10 years. JMIR Mhealth Uhealth 9:e24308. doi: 10.2196/24308, 34287209 PMC8339983

[ref8] Ferry M. (2014). School sports is the solution: what is the problem? Eur. J. Sport Soc. 11, 353–369. doi: 10.1080/16138171.2014.11687972

[ref9] Frison E. Eggermont S. (2020). Toward an integrated and differential approach to the relationships between loneliness, different types of Facebook use, and adolescents’ depressed mood. Commun. Res. 47, 701–728. doi: 10.1177/0093650215617506

[ref10] Gilmore J. N. (2016). Everywear: the quantified self and wearable fitness technologies. New Media Soc. 18, 2524–2539. doi: 10.1177/1461444815588768

[ref11] Gouanvic J. (2005). A Bourdieusian theory of translation, or the coincidence of practical instances: field, ‘habitus’, capital and ‘illusio’. Translator 11, 147–166. doi: 10.1080/13556509.2005.10799196

[ref9003] Guest G. Bunce A. Johnson L. (2006). How many interviews are enough? An experiment with data saturation and variability. Field methods, 18, 59–82.

[ref12] Gui X. Chen Y. Caldeira C. Xiao D. Chen Y. (2017). When fitness meets social networks: investigating fitness tracking and social practices on Werun. Proceedings of the 2017 CHI conference on human factors in computing systems. 1647–1659

[ref13] Hargreaves J. (1986), Sport, Power and Culture. A Social and Historical Analysis of Popular Sports in Britain. Cambridge, UK.

[ref9004] Hargittai E. (2018). The digital reproduction of inequality. In The inequality reader (pp. 660–670). Routledge.

[ref14] Henderson K. A. Bialeschki M. D. Shaw S. M. Freysinger V. J. (1989), A Leisure of One's Own: A Feminist Perspective on Women's Leisure. State College, PA, USA.

[ref15] Hood-Williams J. (1995). Sexing the athletes. Sociol. Sport J. 12, 290–305. doi: 10.1123/ssj.12.3.290

[ref16] Huberty J. Dinkel D. Beets M. W. Coleman J. (2013). Describing the use of the internet for health, physical activity, and nutrition information in pregnant women. Matern. Child Health J. 17, 1363–1372. doi: 10.1007/s10995-012-1160-2 23090284

[ref9005] Juliet M. Corbin S. (2015). Basics of qualitative research: Techniques and procedures for developing grounded theory. SAGE Publications, Incorporated.

[ref17] Kaur H. Singh T. Arya Y. K. Mittal S. (2020). Physical fitness and exercise during the COVID-19 pandemic: a qualitative enquiry. Front. Psychol. 11:590172. doi: 10.3389/fpsyg.2020.590172, 33250827 PMC7673425

[ref18] Kross E. Verduyn P. Sheppes G. Costello C. K. Jonides J. Ybarra O. (2021). Social media and well-being: pitfalls, progress, and next steps. Trends Cogn. Sci. 25, 55–66. doi: 10.1016/j.tics.2020.10.005, 33187873

[ref9009] Lareau A. Weininger E. B. (2003). Cultural capital in educational research: A critical assessment. Theory and society, 32, 567–606.

[ref9006] Lincoln Y. S. Guba E. G. (1988). Criteria for Assessing Naturalistic Inquiries as Reports.

[ref19] Lin S. Liu D. Liu W. Hui Q. Cortina K. S. You X. (2021). Mediating effects of self-concept clarity on the relationship between passive social network sites use and subjective well-being. Curr. Psychol. 40, 1348–1355. doi: 10.1007/s12144-018-0066-6

[ref20] Loprinzi P. D. Day S. Hendry R. Hoffman S. Love A. Marable S. . (2021). The effects of acute exercise on short- and long-term memory: considerations for the timing of exercise and phases of memory. Eur. J. Psychol. 17, 85–103. doi: 10.5964/ejop.2955, 33737976 PMC7957845

[ref21] Markula P. (2003). The technologies of the self: sport, feminism, and Foucault. Sociol. Sport J. 20, 87–107. doi: 10.1123/ssj.20.2.87

[ref22] Martin J. L. (2003). What is field theory? Am. J. Sociol. 109, 1–49. doi: 10.1086/375201

[ref23] Mouatt B. Smith A. E. Mellow M. L. Parfitt G. Smith R. T. Stanton T. R. (2020). The use of virtual reality to influence motivation, affect, enjoyment, and engagement during exercise: a scoping review. Front. Virtual Real. 1:1564664. doi: 10.3389/frvir.2020.564664

[ref25] Nuss K. Moore K. Nelson T. Li K. (2021). Effects of motivational interviewing and wearable fitness trackers on motivation and physical activity: a systematic review. Am. J. Health Promot. 35, 226–235. doi: 10.1177/0890117120939030, 32662277

[ref27] Perreault G. Bell T. R. (2022). Towards a “digital” sports journalism: field theory, changing boundaries and evolving technologies. Commun. Sport 10, 398–416. doi: 10.1177/2167479520979958

[ref28] Rice L. Sara R. (2019). Updating the determinants of health model in the information age. Health Promot. Int. 34, 1241–1249. doi: 10.1093/heapro/day064, 30212852

[ref29] Roth A. Basow S. A. (2004). Femininity, sports, and feminism: developing a theory of physical liberation. J. Sport Soc. Issues 28, 245–265. doi: 10.1177/0193723504266990

[ref9007] Scheerder A. J. Van Deursen A. J. Van Dijk J. A. (2019). Internet use in the home: Digital inequality from a domestication perspective. New media & society, 21, 2099–2118.

[ref30] Sevilmis A. Özdemir İ. García-Fernández J. (2023). The history and evolution of fitness. SPORT TK 12:4. doi: 10.6018/sportk.493851

[ref31] Spaaij R. Farquharson K. Marjoribanks T. (2015). Sport and social inequalities. Sociol. Compass 9, 400–411. doi: 10.1111/soc4.12254

[ref32] Statista. (2024). Digital Fitness & Well-Being—United States. Hamburg, Germany: Statista.

[ref33] Trolan E. J. (2013). The impact of the media on gender inequality within sport. Procedia. Soc. Behav. Sci. 91, 215–227. doi: 10.1016/j.sbspro.2013.08.420

[ref34] Tuveri E. Macis L. Sorrentino F. Spano L. D. Scateni R. (2016). Fitmersive games: fitness gamification through immersive VR. Proceedings of the international working conference on advanced Visual interfaces 212–215

[ref36] Wang X. Wang Y. (2024). Association between digital engagement and urban-rural disparities in Chinese women's depressive symptoms: a national-level cross-sectional study. Digit. Health 10:20552076241239246. doi: 10.1177/20552076241239246, 38577314 PMC10993679

[ref9008] Yong W. (2021). Individual Motivations for Residents’ Cultural Participation and Community Moderation: An Examination Based on Multilevel Linear Models. Library Forum, 41, 56–66.

[ref37] Zuo Y. Ma Y. Zhang M. Wu X. Ren Z. (2021). The impact of sharing physical activity experience on social network sites on residents’ social connectedness: a cross-sectional survey during COVID-19 social quarantine. Glob. Health 17:10. doi: 10.1186/s12992-021-00661-z, 33430894 PMC7797884

